# Interleukin-26–DNA complexes promote inflammation and dermal-epidermal separation in a modified human cryosection model of bullous pemphigoid

**DOI:** 10.3389/fimmu.2022.1013382

**Published:** 2022-10-10

**Authors:** Yuka Mizuno, Sayaka Shibata, Yukiko Ito, Haruka Taira, Eiki Sugimoto, Kentaro Awaji, Shinichi Sato

**Affiliations:** Department of Dermatology, Graduate School of Medicine, The University of Tokyo, Tokyo, Japan

**Keywords:** interleukin-26, DNA, bullous pemphigoid, inflammation, blistering

## Abstract

Bullous pemphigoid (BP) is an autoimmune disease characterized by autoantibody-mediated activation of immune cells and subepidermal blister formation. Excess amounts of extracellular DNA are produced in BP, however, it remains unclear how extracellular DNA contributes to BP pathogenesis. Here we show a possible mechanism by which interleukin (IL)-26 binds to extracellular DNA released from neutrophils and eosinophils to support DNA sensing. Patients with BP exhibited high circulating levels of IL-26, forming IL-26**–**DNA complexes in the upper dermis and inside the blisters. IL-26**–**DNA complexes played a dual role in regulating local immunity and blister formation. First, they enhanced the production of inflammatory cytokines in monocytes and neutrophils. Second, and importantly, the complexes augmented the production and activity of proteases from co-cultured monocytes and neutrophils, which induced BP180 cleavage in keratinocytes and dermal-epidermal separation in a modified human cryosection model. Collectively, we propose a model in which IL-26 and extracellular DNA synergistically act on immune cells to enhance autoantibody-driven local immune responses and protease-mediated fragility of dermal-epidermal junction in BP.

## Introduction

Bullous pemphigoid (BP) is the most common autoimmune blistering disease associated with autoantibodies against BP180 and/or BP230, which are the component proteins of hemidesmosomes (1–4). Basal cells in the lowest layer of the epidermis are tightly bound to the basement membrane *via* hemidesmosomes under homeostasis (4, 5). When autoantibodies accumulate and bind to their target antigens, local complement activation is triggered. Then, inflammatory cells, including neutrophils and monocytes/macrophages, are recruited to the dermal-epidermal junction (DEJ) (6, 7). Subsequently, these activated inflammatory cells, release proteases such as neutrophil elastase (NE) and matrix metalloproteinase-9 (MMP-9), which cleave BP180 in hemidesmosomes (8–11). The series of pathogenic inflammatory responses eventually result in the formation of subepidermal blisters.

In autoimmune diseases, self-derived DNA released from injured cells, either passively or actively, can sense surrounding live cells and trigger inflammation, which contributes to the disease development (12, 13). Small amounts of extracellular DNA are also present in healthy individuals, however, their total amount is regulated by DNase to prevent excessive inflammation (14). In contrast, patients with pathological conditions have high amounts of extracellular DNA in the blood and tissues due to the imbalance of DNA release from cells and its degradation or clearance (15, 16). Previous reports have found that DNA is released from infiltrating eosinophils and neutrophils into the extracellular space and its blood levels are elevated in BP (17). In an *ex vivo* bullous skin model, the stimulation of eosinophils with IL-5 in the presence of BP serum induces dermal-epidermal separation (DES), which is inhibited by the addition of DNase I, indicating the contribution of eosinophil-derived DNA to the BP pathogenesis (18). In addition, neutrophils infiltrating the blistering sites release self-derived DNA and form neutrophil extracellular traps (NETs), which disappear after proper treatment (19). Thus, self-derived DNA released from infiltrating eosinophils or neutrophils has been indeed observed in the lesional skin of patients with BP and contributes to the BP pathogenesis, however, the mechanism by which the extracellular DNA access intracellular DNA sensors to cause blister formation and inflammation has not been elucidated (19).

Interleukin (IL)-26, a cytokine belonging to the IL-20 cytokine subfamily, is a novel inflammatory mediator produced by activated T cells (20–22). In psoriasis and atopic dermatitis, which are T cell-dependent chronic skin inflammatory diseases, IL-26 expression is enhanced in infiltrating T cells of the lesional skin (23, 24). The conventional IL-26 receptor is identified as a heterodimer of IL-20R1 and IL-10R2 (25, 26). IL-20R1 expression is generally restricted in epithelial cells, and most immune cells do not express IL-20R1. Hence, the function of IL-26 on immune cells has long been unknown (25, 26). Recently, IL-26 has been highlighted for its novel property, independent of IL-20R1, which functions as a shuttling protein that links extracellular DNA to inflammation in immune cells (27, 28).

The current study focused on the function of IL-26 as a shuttling protein for extracellular DNA in the development of BP. IL-26–DNA complexes promoted the production of both inflammatory cytokines and proteases from monocytes and neutrophils. As a result, these factors cleaved BP180 in keratinocytes and promoted DES in the skin of patients with BP. Our results provide evidence that IL-26 and DNA synergistically act on immune cells to enhance local immune responses and induce protease-mediated DEJ fragility in BP.

## Results

### Serum cell-free DNA levels are elevated and extracellular DNA is released from neutrophils and eosinophils in BP patients

**Figure 1:**
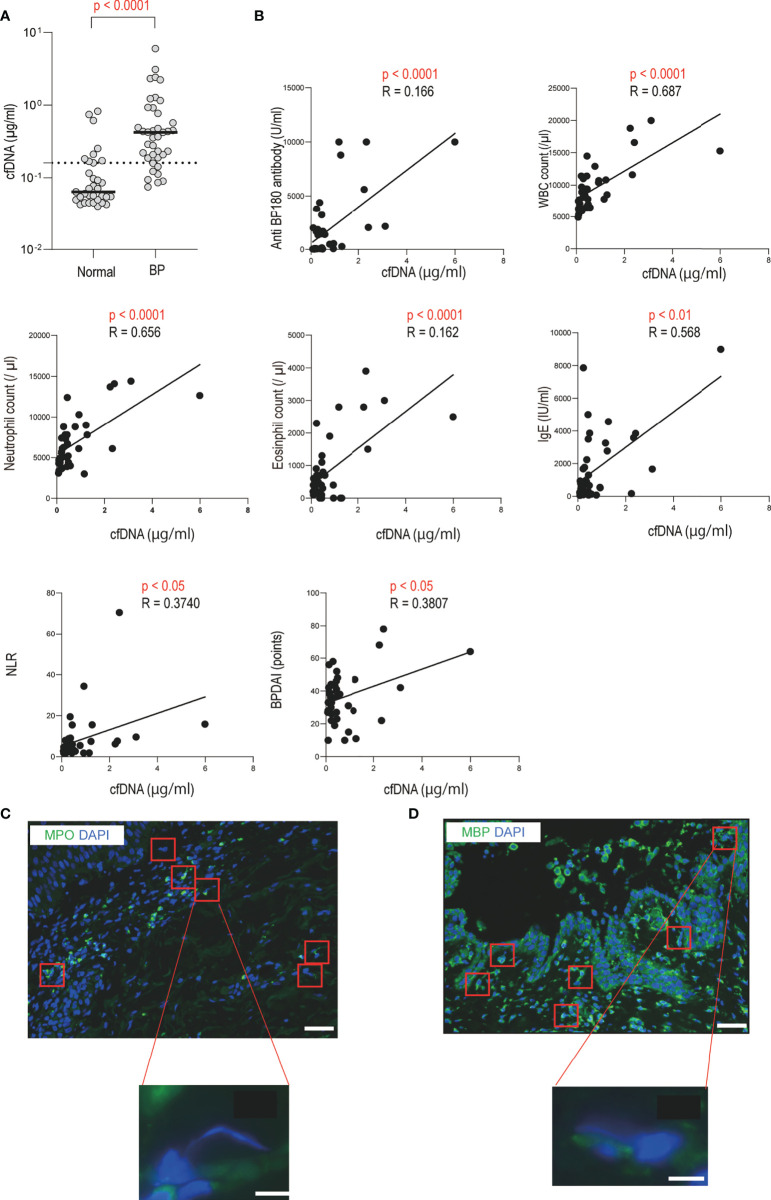
Increased serum cell-free DNA levels and identification of extracellular DNA-producing cells in BP patients **(A)** Serum cell-free DNA levels in BP patients (n = 39), and healthy controls (n = 33) were determined by a specific enzyme-linked immunosorbent assay. The horizontal lines indicate the median values. The broken lines show the cut-off value (mean ± 2 SD of healthy controls). **(B)** Correlations between serum cell-free DNA levels and disease activity markers or BPDAI in BP patients. The solid lines indicate a linear regression line. **(C, D)** Immunofluorescence images for extracellular DNA in the skin sections of BP patients. Extracellular DNA was mostly located within and around subepidermal blisters. DAPI (blue) was used to visualize DNA together with **(C)** MPO (green) or **(D)** MBP (green). Higher magnification images are shown below. Results are representative of six individuals. Scale bar, 50 μm in upper lower magnification, and 5 μm in the lower higher magnification, respectively.

To validate the presence of extracellular DNA in circulation and in local sites of blistering, as shown in previous studies, we initially assessed the circulating cell-free DNA levels of patients with BP and performed DNA staining on skin biopsy specimens (19, 29). Results showed that the serum cell-free DNA levels of BP patients were significantly higher than those of healthy controls (0.422 [0.199-0.846] vs. 0.215 [0.130-0.364] µg/ml, *p* < 0.0001; **Figure 1A**). The cell-free DNA levels were positively correlated with the disease activity score, the Bullous Pemphigoid Disease Area Index (BPDAI) (*p* < 0.05, R = 0.381), and with both serum anti-BP180 antibody (*p* < 0.0001, R = 0.166) and IgE (*p* < 0.01, R = 0.568) levels in BP patients (**Figures 1B, C**). Other variables that showed positive correlation with serum cell-free DNA levels included total white blood cell (*p* < 0.0001, R = 0.687), neutrophil (*p* < 0.0001, R = 0.656) and eosinophil (*p* < 0.0001, R = 0.162) counts in peripheral blood, and neutrophil-to-lymphocyte ratio (NLR) (*p* < 0.05, R = 0.374) (**Figures 1B, C**). All of these factors are known as disease activity biomarkers of BP, and thus, high levels of circulating extracellular DNA are associated with disease progression in BP (30–35). Next, intra and extracellular DNA staining of the BP skin specimens was performed. Morphological changes with elongated DNA filaments, which are indicative of extracellular DNA, were detected inside the blisters and in the upper dermis (**Figures 1C, D**). Positive staining of myeloperoxidase (MPO) and major basic protein (MBP) was observed along and around these filaments (**Figures 1C, D**). Thus, neutrophils or eosinophils primarily produced extracellular DNA in the local inflammatory sites of BP.

### *In vitro* formation of IL-26–DNA complexes and their detection in the skin samples of BP patients

**Figure 2:**
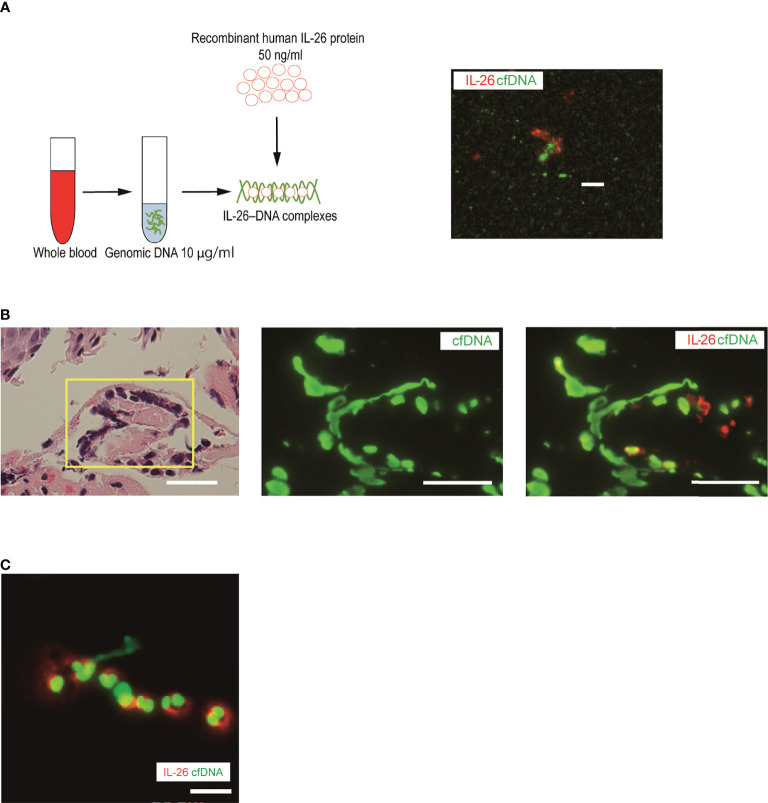
Visualization of IL-26–DNA complexes in the skin and blister fluid from BP patients **(A)** Complexes were formed by mixing human genomic DNA (green, SYTOX Green) with recombinant human IL-26 (red) for 30 minutes. The image shown is representative of three independent experiments. Scale bar, 5 μm. **(B)** Immunofluorescence images for extracellular DNA (green, SYTOX Green) and IL-26 (red) in the subepidermal blisters of skin sections from BP patients. Results are representative of three BP patients. Scale bar, 20 μm. **(C)** Immunofluorescence images for extracellular DNA (green, SYTOX Green) and IL-26 (red) in a blister fluid smear from BP patients. Results are representative of three BP patients. Scale bar, 20 μm.

Overproduction of extracellular DNA does not affect surrounding immune cells because DNA itself cannot reach DNA sensors inside the cells. Extracellular DNA needs to be accompanied by specific carrier proteins to activate immune cells (27, 36). Recent studies have found that one such carrier protein is IL-26 in various pathological conditions, and thus, we next explored the formation of extracellular DNA and IL-26 complexes in the skin samples of BP patients (27, 28, 36–38). First, IL-26–DNA complexes were generated by mixing recombinant human IL-26 with genomic DNA from healthy donors. Positive staining of IL-26 and DNA was detected in an overlapping manner, which is indicative of IL-26–DNA complexes formation (**Figure 2A**). Then, we visualized IL-26–DNA complexes in the skin sections and blister fluid samples from patients with BP. In the same region where chromatin decondensation was observed in hematoxylin-eosin (HE) staining, elongated string-like DNA filaments and surrounding IL-26 stains were detected by immunofluorescence analysis (**Figure 2B**). Similarly, a string-like extension of DNA accompanied by IL-26 staining was also observed in blister fluids from BP patients (**Figure 2C**). These data collectively suggest that IL-26 formed complexes with extracellular DNA at the local pathogenic sites of BP.

### Serum IL-26 levels are elevated and IL-26 is expressed by infiltrating CD3-positive cells in BP patients

**Figure 3:**
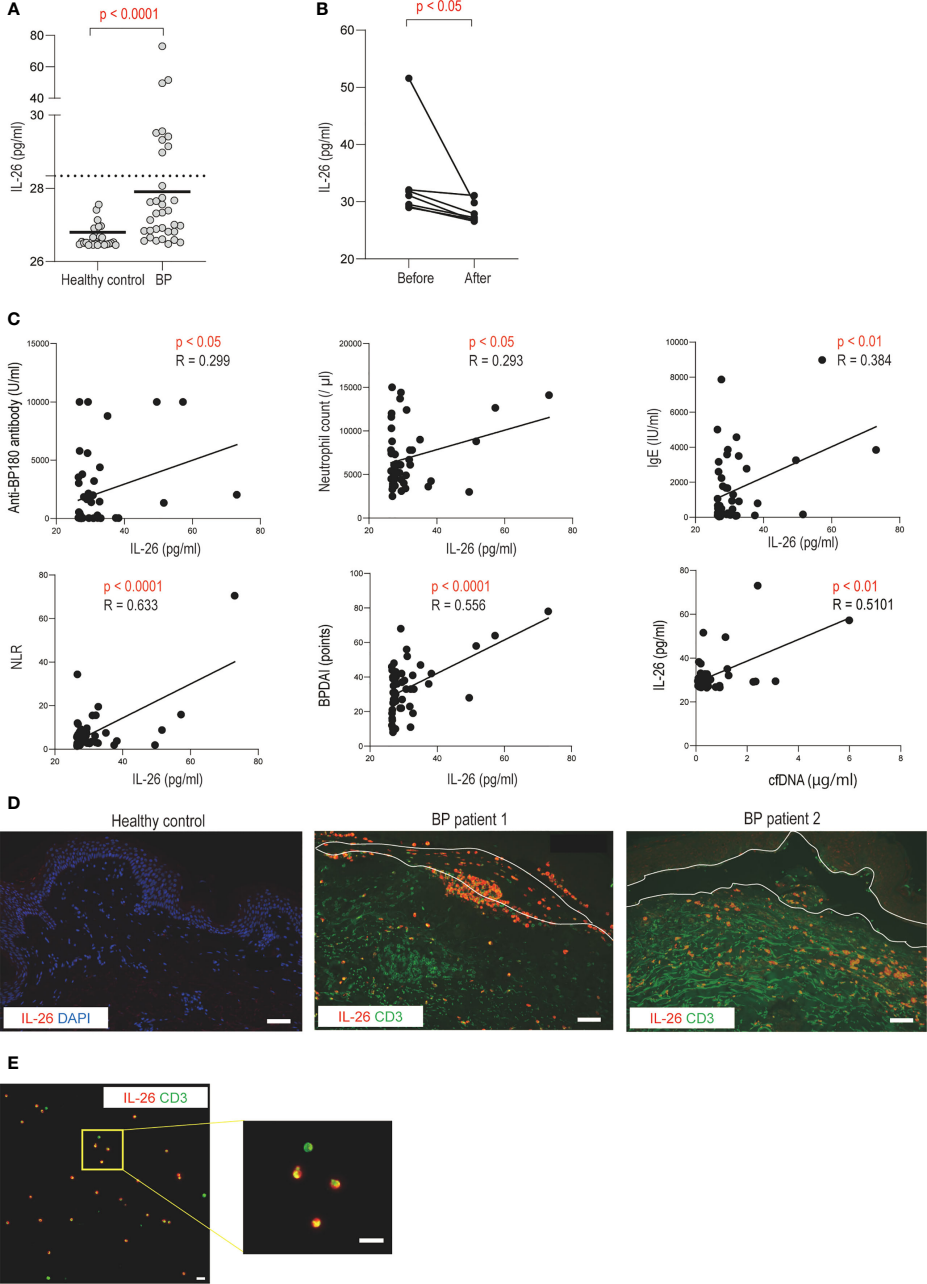
Increased serum IL-26 levels and infiltration of IL-26-producing CD3-positive T cells in BP patients **(A)** Serum IL-26 levels in BP patients (n = 39), and healthy controls (n = 33) were determined by a specific enzyme-linked immunosorbent assay. The horizontal lines indicate the median values. The broken lines show the cut-off value (mean + 2 SD of healthy controls). **(B)** Changes in serum IL-26 levels before and after systemic treatment. **(C)** Correlations between serum IL-26 levels and disease activity markers, BPDAI, or cell-free DNA levels in BP patients. The solid lines indicate a linear regression line. **(D)** Immunofluorescence images for IL-26 in skin sections from healthy controls and BP patients. IL-26 (red) was co-stained with CD3 (green) and DAPI (blue). Results are representative of 2 out of 8 BP patients. Scale bar, 50 μm. **(E)** Immunofluorescence images for IL-26 in a blister fluid smear from BP patients. IL-26 (red) was co-stained with CD3 (green) and DAPI (blue). Higher magnification images are shown on the right side. Results are representative of three BP patients. Scale bar, 20 μm.

Next, we investigated the dynamics of IL-26 in serum and identified IL-26-producing cells in the skin samples from patients with BP. Compared with healthy controls, BP patients exhibited significantly higher serum IL-26 levels (27.906 [26.913-31.909] vs. 26.521 [26.485-26.805] pg/ml, *p* < 0.0001; **Figure 3A**). Serum IL-26 levels were measured in 7 BP patients whose serum samples were available both pre- and post-treatment. Serum IL-26 levels decreased significantly after systemic treatment with improvement in disease activity (*p* < 0.05; **Figure 3B**). Furthermore, serum IL-26 levels in BP patients were positively correlated with serum anti BP180 antibody (*p* < 0.05, R = 0.299) and IgE (*p* < 0.01, R = 0.384) levels and BPDAI (*p* < 0.0001, R = 0.556) (**Figure 3C**). Positive correlations were also detected with neutrophil count in peripheral blood (*p* < 0.05, R = 0.293) and NLR (*p* < 0.0001, R = 0.633) (**Figure 3C**). Importantly, serum IL-26 levels were found to be positively correlated with serum cell-free DNA levels (*p* < 0.01, R = 0.510; **Figure 3C**).

To further investigate IL-26-producing cells in BP, the skin sections from BP patients were co-stained with IL-26 and CD3. CD3-positive cells were detected in the upper dermis and inside the blisters (**Figure 3D**). IL-26 was observed in most CD3-positive cells infiltrating the blisters, and in some cases, even in CD3-positive cells infiltrating the upper dermis (**Figure 3D**). In contrast, no IL-26-positive cells were detected in the normal skin (**Figure 3D**). Consistent with the staining results of skin section samples, most CD3-positive cells were stained with IL-26 in the blister fluid cells from BP patients (**Figure 3E**).

Thus, IL-26 was mainly produced by CD3-positive T cells, and its dynamics are associated with BP progression. Our results that both IL-26 and extracellular DNA were correlated with disease activity and that IL-26–DNA complexes were detected in the lesional skin of BP suggest the potential involvement of these complexes in the pathomechanism of BP.

### IL-26–DNA complexes enhance the expression of proinflammatory cytokines in monocytes and neutrophils

**Figure 4:**
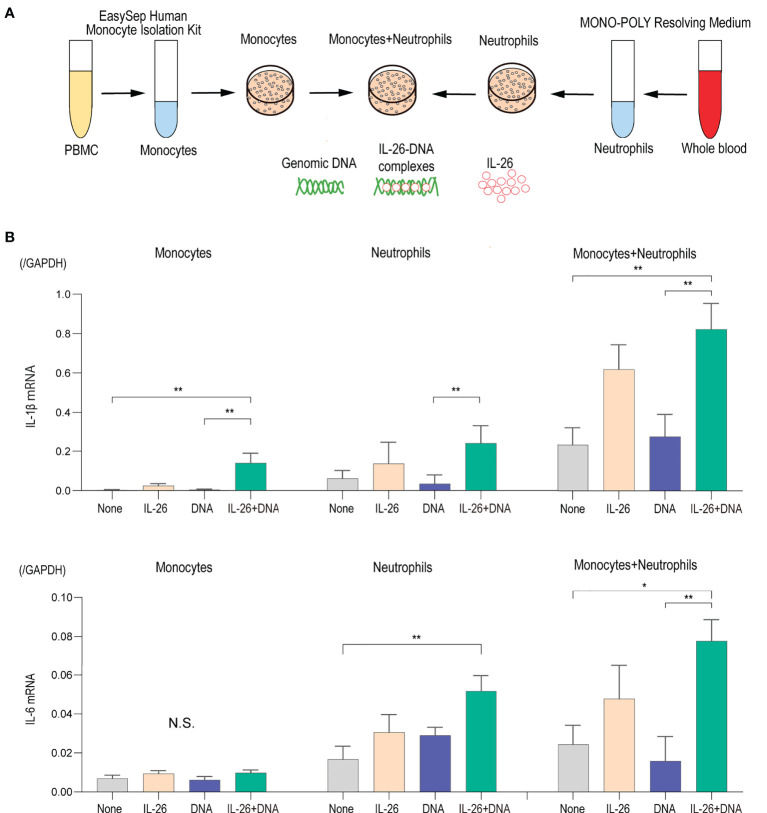
IL-26 synergizes with DNA in inducing proinflammatory cytokines **(A)** Experimental design of the isolation and culture of human monocytes and neutrophils. **(B)** Gene expression levels in monocytes alone, neutrophils alone, and co-cultured monocytes and neutrophils. Cells were stimulated with/without 50 ng/mL of IL-26 and 10 µg/mL genomic DNA or with IL-26–DNA complexes for 6 hours. The relative mRNA expressions of IL-1β and IL-6 were evaluated by quantitative real-time PCR. The results shown are representative of five independent experiments with similar results. Data are presented as mean ± SD. *p < 0.05 **p < 0.001. N.S., Not significant.

To test this hypothesis, we focused on monocytes and neutrophils, which are the predominant cells that infiltrate the DEJ and produce proteases in the blister formation of BP, in order to evaluate the possible role of IL-26–DNA complexes on these cells. First, monocytes and neutrophils were extracted from healthy donors and were cultured either alone or co-cultured. The impact of IL-26–DNA complexes, as well as IL-26 or extracellular DNA alone, was then examined at 6 and 24 hours after stimulation (**Figure 4A**). Stimulation with IL-26 or extracellular DNA alone had no significant effect on IL-1β or IL-6 gene expression in either mono-culture of monocytes and neutrophils or in co-culture of both cells at 6 hours after stimulation (**Figure 4B**). Stimulation with IL-26–DNA complexes significantly enhanced the gene expression of IL-1β in monocytes and that of IL-6 in neutrophils compared to no stimulation (**Figure 4B**). Importantly, the co-culture of monocytes and neutrophils increased baseline cytokine expression, which was further enhanced by stimulation with IL-26–DNA complexes (**Figure 4B**). We also examined the expression levels of IL-1β and IL-6 at 24 hours after stimulation. Similar to the results at 6 hours after stimulation, there was a trend toward a significant increase in cytokine expression of mono-culture of monocytes or neutrophils stimulated by IL-26–DNA complexes compared to those without stimulation, however, the difference was less pronounced (**Supplementary Figure 1**). There was no enhancement of baseline cytokine expression induced by co-incubation of monocytes and neutrophils, which may be attributed to the short survival of neutrophils (**Supplementary Figure 1**) (39). Neutrophils may have lost their interaction with monocytes due to the reduced activity of neutrophils at 24 hours after the beginning of co-culture.

### IL-26–DNA complexes enhances the production and activity of proteases in monocytes and neutrophils

**Figure 5:**
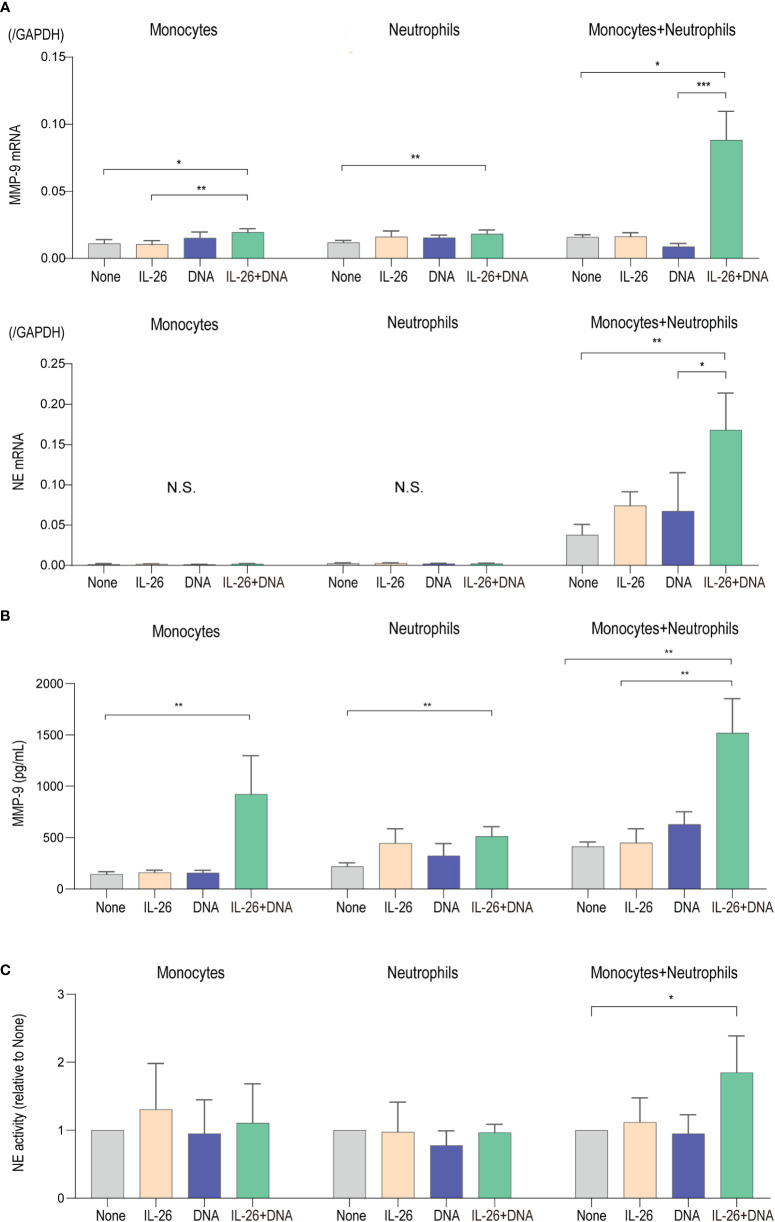
IL-26 synergizes with DNA in enhancing the production and activity of proteases **(A)** Gene expression levels in monocytes alone, neutrophils alone, and co-cultured monocytes and neutrophils. Cells were stimulated with/without 50 ng/L of IL-26 and 10 µg/mL genomic DNA or with IL-26–DNA complexes for 6 hours. The relative mRNA expressions of MMP-9 and NE were evaluated by quantitative real-time PCR. Results shown are representative of five independent experiments with similar results. Data are presented as mean ± SD. *p < 0.05 **p < 0.001 ***p < 0.0001. N.S., Not significant. **(B)** Protein levels of MMP-9 and **(C)** NE activities of supernatants from monocytes alone, neutrophils alone, and co-cultured monocytes and neutrophils. NE activity scores are presented relative to none. Data are presented as mean ± SD. *p < 0.05 **p < 0.001 ***p < 0.0001. N.S., Not significant.

Eventual blister formation in BP is caused by proteases that cleave BP180 and induce loss of dermal-epidermal adhesion (8–11, 18, 40). To further explore the impact of IL-26–DNA complexes on the step of blister formation, we analyzed the expression levels of MMP-9 and NE, the two major proteases involved in the cleavage of BP180, in monocytes and/or neutrophils stimulated with either IL-26 or DNA alone, or with IL-26**–**DNA complexes. MMP-9 mRNA expression was significantly upregulated by stimulation with IL-26–DNA complexes compared to no stimulation in mono-culture of monocytes and neutrophils, although stimulation with IL-26 or DNA alone did not affect the MMP-9 gene expression (**Figure 5A**). Co-culture of monocytes and neutrophils dramatically enhanced the MMP-9 expression by stimulation with IL-26–DNA complexes (**Figure 5A**). NE mRNA expression was below detectable levels in mono-cultures of monocytes or neutrophils. NE mRNA was detected under the co-culture of monocytes and neutrophils, and its expression was significantly increased by stimulation with IL-26–DNA complexes (**Figure 5A**). MMP-9 protein concentrations and NE activity levels were also determined using the supernatants from monocyte and/or neutrophil culture at 6 hours. Consistent with the gene expression results, MMP-9 protein concentrations in co-culture were significantly elevated by stimulation with IL-26–DNA complexes compared to no stimulation (**Figure 5B**). NE activity in co-culture was also significantly higher with IL-26–DNA complexes stimulation compared to no stimulation (**Figure 5C**).

Taken together, the synergistic effects of IL-26 and DNA on monocytes and neutrophils shown here have strongly supported their functional enhancement in the regulation of inflammation and protease activation that occurs locally in BP. Although both functions on monocytes or neutrophils alone were limited, these effects were dramatically enhanced by the cellular interaction of monocytes and neutrophils. Thus, the interaction between immune cells, along with complex formation by IL-26 and extracellular DNA, may be an important event in the development of BP.

### IL-26–DNA complexes induce MMP-9- and NE-dependent loss of hemidesmosome protein BP180 expression and *in vitro* cell detachment

**Figure 6:**
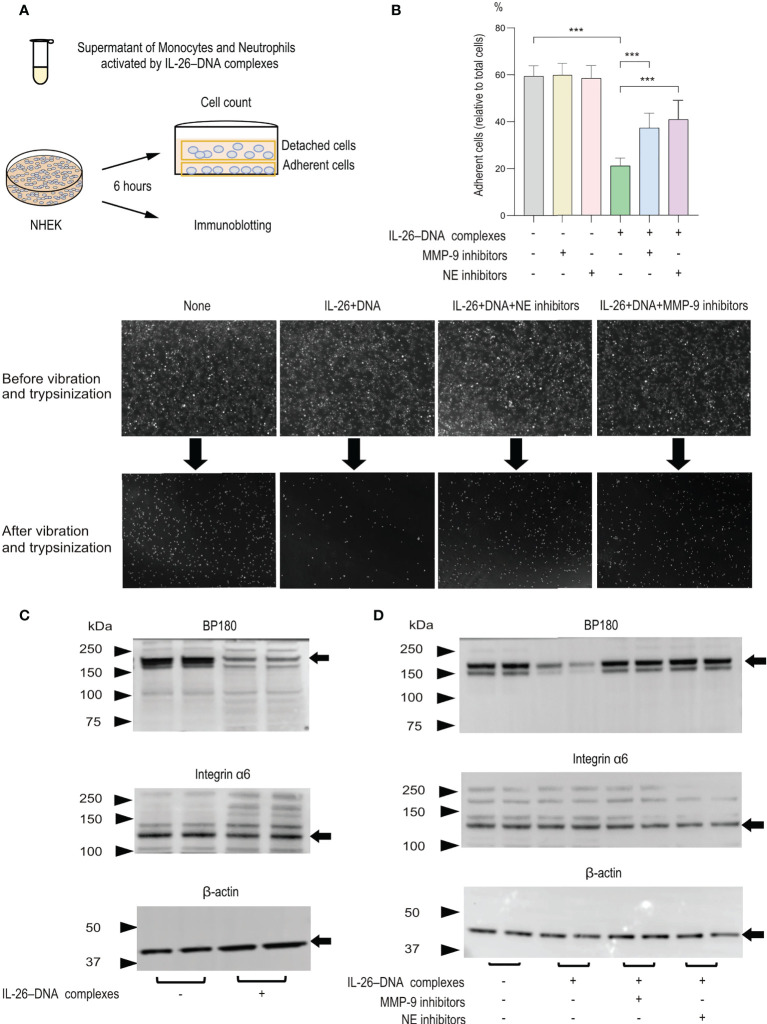
Effects of monocytes and neutrophils stimulated with IL-26–DNA complexes on cultured NHEKs *in vitro* **(A)** Experimental design for analyzing detachment of NHEKs and cleavage of hemidesmosomal protein BP180. **(B)** The detachment degree of NHEKs from culture plates was assessed after 6 hours of incubation with medium including the supernatants from monocytes and neutrophils treated with vehicle or IL-26–DNA complexes. MMP-9 or NE inhibitors were pre-administered in both conditions. The ratio of the adherent cells to total cells (adherent cells + floating cells) in each condition was calculated. Results shown are representative of three independent experiments with similar results. Data are presented as mean ± SD. ***p < 0.0001. The images shown are NHEKs incubated with medium containing supernatants treated with vehicle or IL-26–DNA complexes, or with medium containing supernatants treated with IL-26–DNA complexes along with MMP-9 or NE inhibitors. The supernatants were collected from co-cultured monocytes and neutrophils **(C, D)** Representative immunoblotting images of BP180, integrin α6, and β-actin protein expression in NHEKs. Results shown are representative of three independent experiments with similar results. Black arrows indicate the bands for BP180, integrin α6 and β-actin. Molecular weight (MW) is indicated as black triangles on the left sides. Cell lysates of NHEKs incubated with medium containing supernatants of monocytes and neutrophils were subjected to immunoblotting analysis. Monocytes and neutrophils were treated with vehicle or IL-26–DNA complexes **(C)** or were pre-administered MMP-9 or NE inhibitors before treatment with complexes **(D)**.

We next aimed to elucidate the mechanisms of IL-26–DNA complexes-mediated blister formation in the pathogenesis of BP. For this purpose, we collected supernatants from the co-culture of monocytes and neutrophils stimulated with or without IL-26–DNA complexes and performed an experimental system in which normal human epidermal keratinocytes (NHEKs) were stimulated with these supernatants (**Figure 6A**). We first examined changes in the attachment of NHEKs induced by IL-26–DNA complexes-stimulated supernatants. After vibration and trypsinization, the number of cells attached to the bottom of culture plates and cells released in the medium was counted, and the ratio of adherent cells to total cells (adherent cells + floating cells) was calculated. The adhesion of NHEKs to culture plates was reduced to 60% after vibration and trypsinization. Treatment with medium containing IL-26–DNA complexes-stimulated supernatants further reduced the number of adherent cells by approximately one-third compared to treatment with a medium containing vehicle-stimulated supernatants (**Figure 6B**). When NHEKs were treated along with MMP-9 or NE inhibitors, the number of adherent cells was restored significantly up to two-thirds of that treated with a medium containing vehicle-stimulated supernatants, suggesting that the cell detachment induced by IL-26–DNA complexes is partially MMP-9- or NE-dependent.

We next examined protein expression levels of BP180, integrin α6, and β-actin by immunoblotting. Consistent with reduced adherence to the culture plate, NHEKs treated with a medium containing IL-26–DNA complexes-stimulated supernatants exhibited a clear decrease in BP180 expression compared to those treated with a medium including vehicle-stimulated supernatants (**Figure 6C**). In contrast, the protein levels of integrin α6 and β-actin were comparable between NHEKs treated with IL-26–DNA complexes- and vehicle-stimulated supernatants (**Figure 6C**). To confirm whether the decreased expression of BP180 is dependent on MMP-9 or NE activity, monocytes and neutrophils were incubated with MMP-9 or NE inhibitors before administration of IL-26–DNA complexes. Results showed that NHEKs stimulated with supernatant containing MMP-9 or NE inhibitors restored BP180 expression, indicating that BP180 cleavage is dependent on the activity of MMP-9 and NE induced by IL-26–DNA complexes in monocytes and neutrophils (**Figure 6D**).

### IL-26–DNA complexes-treated monocytes and neutrophils induced DES in a human cryosection model of BP

**Figure 7:**
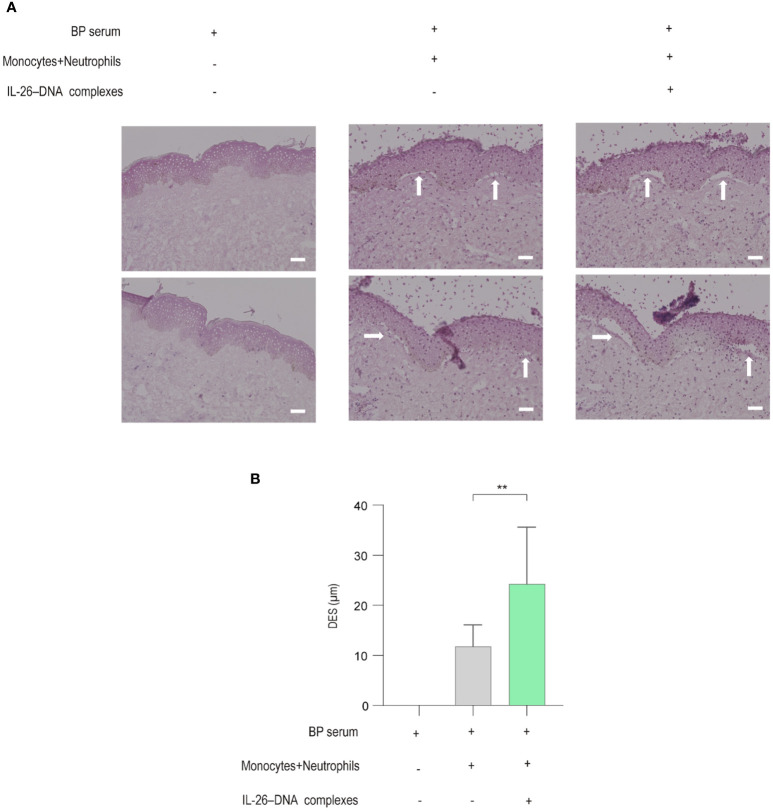
Effects of monocytes and neutrophils stimulated with IL-26–DNA complexes in an *ex vivo* BP skin model **(A)** Representative images showing DES (white arrows) on cryosections in the presence of BP serum incubated with/without monocytes and neutrophils stimulated with/without IL-26–DNA complexes. **(B)** DES lengths measured on cryosections. Data are presented as mean ± SD. **p < 0.001.

We finally utilized an *ex vivo* BP model to examine whether IL-26–DNA complexes promote DES. Frozen sections of healthy normal skin were initially used and were incubated with BP serum together with monocytes and neutrophils. DES was barely induced in healthy normal skin, and therefore, we attempted to induce DES using erythematous areas from the BP skin as a modified model in this study. Incubation of BP erythematous skin with BP serum, and subsequently with monocytes and neutrophils stimulated by IL-26–DNA complexes or vehicle, resulted in the induction of DES (**Figure 7A**). The BP erythematous skin incubated with serum from the same BP patient without monocytes and neutrophil incubation were used as a negative control (**Figure 7A**). In BP erythematous skin incubated with BP serum and vehicle-treated monocytes and neutrophils, DES was only slightly observed (**Figure 7A**). In contrast, DES was well induced in cryosections incubated with BP serum in the presence of IL-26–DNA complexes-stimulated monocytes and neutrophils (**Figure 7A**). The actual distances of DES in the IL-26**–**DNA complexes-stimulated group were significantly longer compared to those in the vehicle-treated group, although DES was not observed in cryosections incubated with BP serum alone (**Figure 7B**).

## Discussion

**Figure 8:**
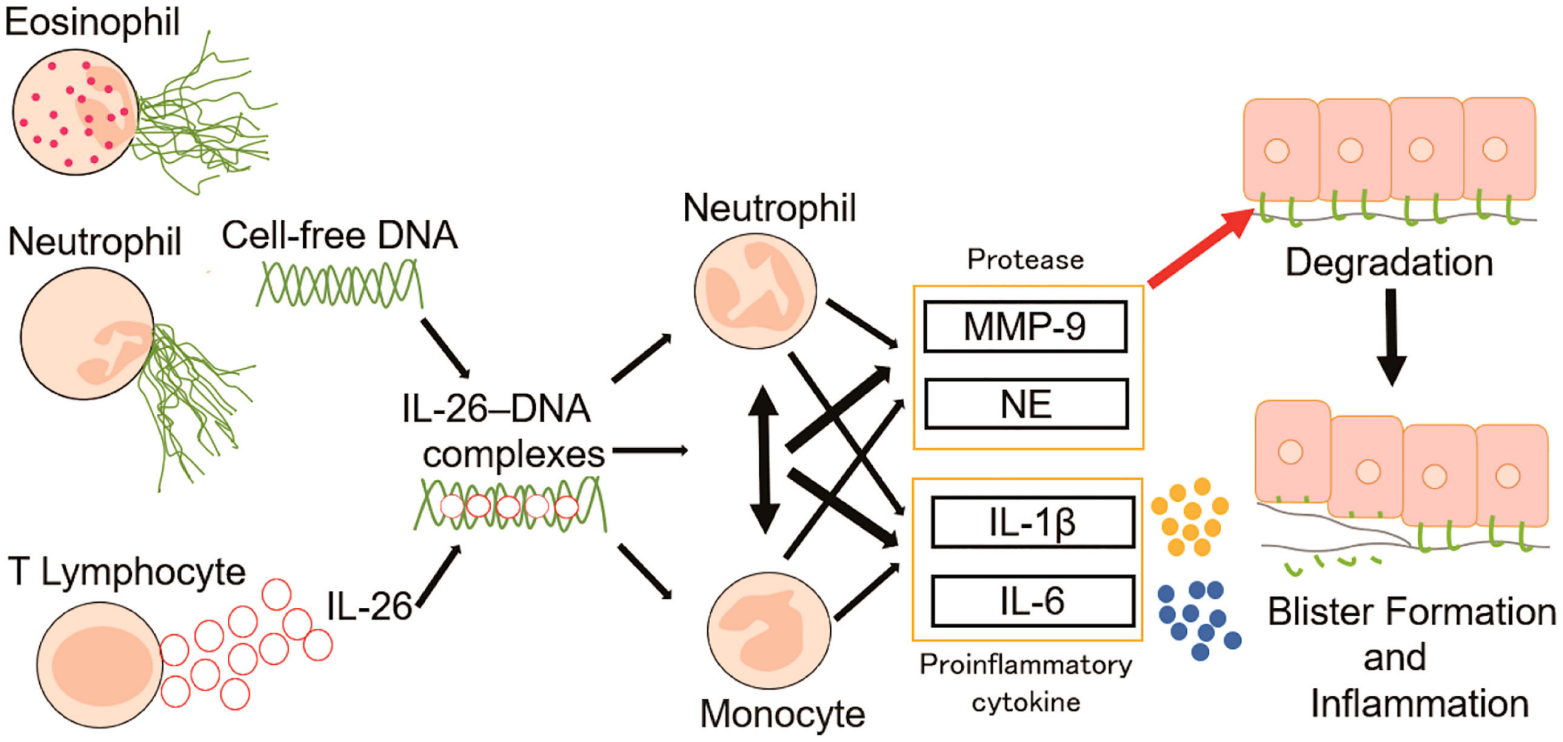
Proposed model for the role of IL-26–DNA complexes in BP pathology. Extracellular DNA released from infiltrating eosinophils and neutrophils form complexes with IL-26 derived from CD3-positive lymphocytes. The resulting IL-26–DNA complexes trigger the production of proteases (MMP-9 and NE) and proinflammatory cytokines (IL-1β and IL-6) from monocytes and/or neutrophils. These proteases and proinflammatory cytokines promote degradation of hemidesmosomal protein BP180, resulting in the formation of subepidermal blisters.

Extracellular DNA is reported to be abundant both in the blood and in the skin of patients with BP (19, 29). To elucidate how extracellular DNA exerts its function at the local sites of BP skin, the present study spotlighted the emerging cytokine IL-26, which serves as a shuttling protein for extracellular DNA (27, 28, 41). Our results showed that patients with BP had higher levels of both IL-26 and cell-free DNA in the circulation compared with healthy controls. Moreover, IL-26 was observed to bind to extracellular DNA in the regions infiltrated by inflammatory cells in the blisters and the upper dermis of the BP skin. Previous reports have shown that IL-26**–**DNA complexes exhibit immunostimulatory effects *via* the IFN gene-stimulating factor (STING) and other pathways, following access to intracellular DNA sensors (42). Therefore, we first examined whether these complexes promote the production of inflammatory cytokines in neutrophils and monocytes. We found that the expression of inflammatory cytokines such as IL-1β and IL-6 were indeed increased in monocytes and neutrophils upon stimulation with the IL-26**–**DNA complexes, but even more striking was the upregulated expression of MMP-9 and NE. The production of these proteases acted to decrease the expression of BP180 in epidermal keratinocytes. The pathological function of the IL-26**–**DNA complexes was finally confirmed by an *ex vivo* human BP model that mimics the actual pathogenesis of BP. The local pathogenesis of BP is initiated by the binding of anti-BP180 IgG autoantibodies to their target antigen at the DEJ (43). The autoantibodies bound to the target antigen BP180 contribute to blister formation *via* two mechanisms: complement-dependent and -independent pathways. Complement activation is accompanied by immune cell accumulation at the DEJ and subsequent release of proteases in complement-dependent pathways, while BP180 is internalized by basal cells and depleted in complement-independent pathways (43–46). The function of the IL-26**–**DNA complexes demonstrated in the present study is associated with potentiating the former mechanism *via* enhancement of local inflammation and protease production (**Figure 8**).

Both cell-free DNA and IL-26 levels were elevated in patients with BP in circulation and their levels correlated with serum biomarkers reflecting the disease activity of BP. However, even if excess amounts of DNA exist in an extracellular environment, released DNA by itself cannot activate immune cells because of the inaccessibility to the intracellular DNA sensors (27, 36). Extracellular DNA crosses membranes and becomes accessible to DNA sensors only when accompanied by shuttling proteins, including IL-26 (27, 36). Regarding the function of IL-26, IL-20R1 is a component of the heterodimeric receptor for IL-26 and is expressed in limited cell types such as epithelial cells (25, 26). Recent reports indicate that myeloid cells also express IL-20R1 mRNA under some conditions, but its effect is limited, and the mechanism by which IL-26 affects immune cell function is assumed to be independent of IL-20R1 (28). In the present study, the cytokine production from monocytes and/or neutrophils was not enhanced by DNA alone and was only slightly enhanced by IL-26 alone. As shown by our current findings and other studies, extracellular DNA cooperates with the carrier protein IL-26 to exert strong immunostimulatory effects on neutrophils and/or monocytes (27, 28, 38). Circulating IL-26 and cell-free DNA levels were positively correlated, indicating the possibility that both factors may affect each other directly or indirectly. IL-26**–**DNA complexes-induced monocyte activation may lead to further activation of IL-26-producing T cells and release of DNA from neutrophils and eosinophils at the sites of tissue damage, which may increase the total amount of IL-26**–**DNA complexes. Such an inflammatory feed-forward circuit may underlie the chronicity of BP.

The function of IL-26 as promoting DNA sensing in an immunostimulatory manner has been attracting attention in the field of pathological conditions regarding chronic inflammatory disorders or infectious diseases (27, 28, 36–38). The production of cytokines or chemokines, such as IFN, IL-6, IL-1β, and CXCL8, is enhanced in immune cells stimulated by IL-26**–**DNA complexes compared with stimulation by IL-26 or DNA alone (28, 38). In agreement with these previous reports, the expression of IL-1β and IL-6 was the highest when monocytes and/or neutrophils are stimulated with both IL-26 and DNA. Importantly, we report here novel findings that IL-26**–**DNA complexes also promote the production of proteases, specifically MMP-9 and NE, which contribute to the direct and ultimate formation of subepidermal blisters in BP (8–11). MMP-9 is not exclusively produced by monocytes or neutrophils. Eosinophils and mast cells, both of which are highly infiltrative and important inflammatory mediators in BP, also produce MMP-9 (47, 48). TNF-α and/with IL-3 have been reported to induce the synthesis of MMP-9 in eosinophils (49, 50). It could be assumed that IL-26 functions as a transport molecule for extracellular DNA, as it does in monocytes and neutrophils (36). IL-26**–**DNA complexes may become another new activator for the production of MMP-9 in eosinophils and mast cells. IL-26**–**DNA complexes have been reported to activate intracellular innate cytoplasmic receptors, STING (42). The STING signaling phosphorylates interferon regulatory factor 3 (IRF3) and nuclear factor kappa B (NF-κB) inhibitor IκBα, which results in translocation of NF-κB to the nucleus (51). Given that MMP-9 as well as IL-1β and IL-6 is one of the target genes of the NF-κB transcription factor, the simultaneous induction of inflammatory cytokine and protease expression may be dependent on a series of STING activation.

We found that the co-culture of monocytes and neutrophils dramatically increased the expression of cytokines and proteases stimulated by IL-26**–**DNA complexes, compared to monocytes or neutrophils cultured alone. Previous studies have shown that the interaction between monocytes and neutrophils enhances the release of mediators and the ultimate formation of subepidermal blisters, as reported by Graauw and Sitaru et al. (18). Our results were consistent with their results, as NE expression was detected only under the co-culture condition of monocytes and neutrophils. Thus, immune cell interaction and activation are essential for the development and maintenance of BP, supporting the importance of suppressing immune activation and intracellular crosstalk during the treatment.

This study has a potential limitation. The direct evidence that the IL-26**–**DNA complexes contribute to BP development *in vivo* is lacking. BP180-humanized or NC16A-humanized mice can be used to explore the impact of specific factors in BP pathogenesis or to demonstrate drug effects *in vivo* (52–54). Although IL-26 is not produced in mice or rats, mice express a functional IL-26 receptor, and administration of human IL-26 has been reported to affect the severity of experimental autoimmune encephalomyelitis (55, 56). Further *in vivo* experiments using BP animal models are desirable to confirm the findings obtained from human *in vitro* and *ex vivo* experiments in this study.

In conclusion, our studies have provided a potential contribution of extracellular DNA *via* its shuttling protein IL-26 in the development of BP. Strategies for controlling the inflammation and vulnerability of DEJ in BP may be further developed by modulating IL-26 function.

## Methods

### Patients

A retrospective analysis was performed on patients with BP who visited the University of Tokyo Hospital (Tokyo, Japan) from January 2008 to December 2021. A diagnosis of BP was made based on the combination of typical clinical features (tense blisters with or without urticarial erythema), histopathological findings (subepidermal separation with or without eosinophilic infiltration), linear deposition of IgG and/or C3 at the DEJ determined by direct and indirect immunofluorescent microscopy, IgG deposits at the epidermal side detected by indirect immunofluorescence microscopy on salt-split-skin, and detection of serum BP180- and/or BP230specific IgG autoantibodies.

Clinical and hematological data were collected from the patient’s medical records upon diagnosis. The Bullous Pemphigoid Disease Area Index (BPDAI) was scored for the assessment of disease severity (30). The hematological findings collected were serum titers of anti-BP180 autoantibodies, and a count of white blood cells, neutrophils, lymphocytes, and eosinophils in peripheral blood.

### Samples collected from patients with BP and healthy controls

Serum samples were collected from 48 patients and sex/age-matched 33 healthy controls after obtaining written informed consent (**Supplementary Table 1**). Patients with BP who had taken dipeptidyl peptidase-4 (DPP4) inhibitors before disease onset of the disease were not included in this study. None of the patients had received systemic treatment with prednisolone or immunosuppressive drugs before sample collection. The healthy controls had no history of allergy or skin diseases, including atopic dermatitis and psoriasis. Serum, blister fluid, and skin samples were stored at -80°C before use. The medical ethics committee of the University of Tokyo approved all described studies (No. 0695) and the study was conducted according to the principles of the Declaration of Helsinki.

### Isolation of human neutrophils and monocytes and their cell culture

Primary human monocytes were isolated from the primary blood mononuclear cells (PBMCs) of healthy donors using EasySep™ Human Monocyte Enrichment Kit (STEMCELL Technologies, Grenoble, France), according to the manufacturer’s instruction. Primary human neutrophils were isolated from the whole blood of healthy donors using a Mono-Poly resolving medium (DS Pharma Biomedical, Osaka, Japan). Isolated monocytes and neutrophils were cultured in RPMI-1640 (GIBCO, Grand Island, NY) supplemented with 10% FBS at 37°C for 6 or 24 hours. In cryosection assays, isolated monocytes and neutrophils were co-cultured at a density of 1:2 (18).

### Cell culture of NHEKs

Primary NHEKs (Kurabo, Osaka, Japan) were cultured in EpiLife medium (Thermo Fisher Scientific, Waltham, MA) with supplements (penicillin G sodium, streptomycin sulfate, and amphotericin B) in 5% CO2 at 37°C. NHEKs were cultured to obtain subconfluent, with approximately 70% confluence, just before the experiments.

### Genomic DNA andIL-26–DNA complexes preparation

Genomic DNA was extracted from healthy donors using the Genomic DNA Clean & Concentrator Kit (Zymo Research, Irvine, CA, USA). DNA concentration was measured using the Qubit fluorimeter (Life Technologies, Carlsbad, CA). IL-26**–**DNA complexes were formed by incubating recombinant human IL-26 (R&D Systems, Minneapolis, USA) at a concentration of 50 ng/mL with 10 µg/mL genomic DNA for 30 min at 37˚C.

### Immunofluorescence staining

Formalin-fixed paraffin-embedded skin sections were deparaffinized and permeabilized with 0.2% Triton-X 100 in PBS. After heat-induced antigen retrieval with a citrate-based antigen unmasking solution (Vector Laboratories, Burlingame, California, US), sections were blocked with 5% BSA and 5% normal goat serum in PBS for 1 hour at room temperature. The sections were then incubated with primary antibodies against MPO (R&D Systems, Minneapolis, USA), MBP(Cloud Clone, Houston, TX), IL-26 (Abcam, Cambridge, UK), and CD3 (Abcam, Cambridge, UK) at 4℃ overnight, followed by 2 hours of incubation at room temperature with matched secondary antibodies (Abcam, Cambridge, UK). DNA staining and section mounting were performed using DAPI Fluoromount-G (Southern Biotechnology Associates, Birmingham, AL, USA) or SYTOX green Nucleic Acid Stain (1 µM; Thermo Scientific, Waltham, MA). Immunofluorescent images were obtained using an all-in-one fluorescence microscope (BZ-X810, Keyence, Itasca, IL, USA). Image analysis was performed by using BZ-X800 Analyzer software version 1.1.1.8 (Keyence, Itasca, IL, USA).

### Enzyme-linked immunosorbent assay and neutrophil elastase activity assay

The protein concentration of serum and cell culture supernatants were examined using enzyme-linked immunosorbent assay (ELISA). The ELISA kits for cell-free DNA (Active Motif, Carlsbad, CA, USA), IL-26 (Cloud Clone, Houston, TX, USA), and MMP-9 (Proteintech, Deansgate Manchester, UK) were used. All experiments were performed according to the manufacturer’s instructions. The activity of monocytes and/or NE release was performed using the Elastase Activity Assay kit (Abcam, Cambridge, UK) according to the manufacturer’s instruction.

### SDS-polyacrylamide gel electrophoresis and immunoblotting

Proteins from keratinocytes were extracted using RIPA lysis buffer (Santa Cruz Biotechnology, Santa Cruz, CA, USA). Proteins were separated by SDS-polyacrylamide gel electrophoresis and transferred to the Immobilon-P transfer membrane (Millipore, Bedford, MA, USA). The membrane was blocked in 5% non-fat milk buffer for 1 hour at room temperature and then incubated with primary antibodies against β-actin (Santa Cruz Biotechnology, Santa Cruz, CA, USA), COL17 (biorbyt, Cambridge, UK), and Integrin alpha 6 (Proteintech, Deansgate Manchester, UK) at 4°C overnight, followed by incubation with the appropriate secondary antibodies (Proteintech, Deansgate Manchester, UK) for 1 hour at room temperature. Visualization was performed with enhanced chemiluminescence technology (Thermo Scientific, Rockford, IL, USA).

### RNA isolation and quantitative real-time PCR

Total RNA was isolated from monocytes or/and neutrophils using Direct-zol RNA Microprep (Zymo Research, Irvine, CA, USA) and cDNA was synthesized using ReverTra Ace qPCR RT Master Mix (TOYOBO, Osaka, Japan). Gene expression was quantified using THUNDERBIRD SYBR qPCR Mix (TOYOBO) or TaqMan Fast Advanced Master Mix (Thermo Scientific, Rockford, IL, USA). GAPDH gene expression was used as an internal control and the relative expression levels of each gene were determined by the 2^−△△CT^ method. The primer sequences used were listed in **Supplementary Table 2**.

### Detachment assay of NHEKs

Subconfluent NHEKs in a 6-well plate (Iwaki, Tokyo, Japan) were incubated for 6 hours with medium including the supernatants from IL-26**–**DNA complexes- or vehicle-stimulated monocytes and neutrophils. Cells were vortexed at 700 rpm/min for 30 minutes, followed by 0.25% trypsinization for 5 minutes at 37℃. After releasing cells completely into the medium by pipetting, the number of cells attached to the bottom of culture plates and cells released in the medium was counted using a blood cell counter under a microscope. In some experiments, NHEKs were treated with MMP-9 or NE inhibitors (both from Abcam, Cambridge, UK) for 30 minutes before supernatant administration from IL-26**–**DNA- or vehicle-stimulated monocytes and neutrophils.

### Cleavage assay for BP180

Subconfluent NHEKs in a 6-well plate (Iwaki, Tokyo, Japan) were incubated for 6 hours with medium including the supernatants from IL-26**–**DNA complexes- or vehicle-treated monocytes and neutrophils in duplicate. After incubation, NHEKs were analyzed by SDS-polyacrylamide gel electrophoresis and immunoblotting. In some experiments, monocytes and neutrophils were treated with MMP-9 or NE inhibitors (both from Abcam, Cambridge, UK) for 30 minutes prior to IL-26**–**DNA complexes administration, and NHEKs incubated in medium containing their supernatant were used for the analysis. Visualization was performed with enhanced chemiluminescence technology (Thermo Scientific, Rockford, IL, USA).

### Cryosection assay

Cryosection assay was performed as previously reported with minor modifications (57–59). Frozen section specimens were selected from erythematous areas peripheral to the bullous margin of BP patients, and BP sera were selected from patients with high serum anti-BP180 antibody levels (> 1000 U/mL). The BP patient serum (BPS) diluted 1:2 with PBS to 600 µl was applied to the skin sections and incubated for 2 hours at 37°C. Monocytes (5.0 × 10^6^ cells/mL) and neutrophils (1.0 × 10^7^ cells/mL) stimulated by IL-26**–**DNA complexes or vehicle were subsequently added to the skin sections for further incubation of 4 hours at 37°C. The skin sections were then fixed with 3.7% formalin and stained with hematoxylin and eosin. DES images were obtained using an all-in-one fluorescence microscope (BZ-X810, Keyence, Itasca, IL, USA). The total lengths of DES were measured using BZ-X800 Analyzer software version 1.1.1.8 (Keyence, Itasca, IL, USA).

### Statistical analysis

The data are presented as the median ± standard deviation. Statistical calculations were performed using GraphPad Prism version 8 (GraphPad Software Inc, San Diego, USA). For human sample analyses, unpaired Mann-Whitney U test or paired Wilcoxon signed-rank test for two-group comparison, and Spearman’s rank correlation coefficient for two continuous variables were used. For other *in vitro* or *ex vivo* experiments, a two-tailed *t*-test for two-group comparison and one-way analysis of variance with Dunn’s *post hoc* test for multiple group comparison were utilized. Significant differences are illustrated as *p < 0.05, **p < 0.001, ***p < 0.0001.

## Data availability statement

The original contributions presented in the study are included in the article/**Supplementary Materials**. Further inquiries can be directed to the corresponding author.

## Ethics statement

The studies involving human participants were reviewed and approved by medical ethics committee of the University of Tokyo (No. 0695). The patients/participants provided their written informed consent to participate in this study.

## Author contributions

YM, SSh, and SSa designed the experiments and wrote the manuscript. YM performed most of the experiments and analyzed the data, with contributions from YI, HT, ES, and KA. All authors discussed the results and commented on the manuscript. All authors contributed to the article and approved the submitted version.

## Acknowledgments

We thank M. Enomoto, M. Hanzawa, and N. Watanabe for technical support. There was no specific funding for this work.

## Conflict of interest

The authors declare that the research was conducted in the absence of any commercial or financial relationships that could be construed as a potential conflict of interest.

## Publisher’s note

All claims expressed in this article are solely those of the authors and do not necessarily represent those of their affiliated organizations, or those of the publisher, the editors and the reviewers. Any product that may be evaluated in this article, or claim that may be made by its manufacturer, is not guaranteed or endorsed by the publisher.

## Supplementary material

Supplementary Figure 1 Gene expression levels in monocytes alone, neutrophils alone, and co-cultured monocytes and neutrophils 24 hours after stimulation Cells were stimulated with/without 50 ng/mL of IL-26 and 10 µg/mL genomic DNA or with IL-26–DNA complexes for 24 hours. The relative mRNA expressions of MMP-9, IL-1β, and IL-6 were evaluated by quantitative real-time PCR. Data are presented as mean ± SD. **p* < 0.05***p* < 0.001****p* < 0.0001.

## Abbreviations

BP, bullous pemphigoid; DEJ, dermal-epidermal junction; NE, neutrophil elastase; MMP, matrix metalloproteinase; IL-26, interleukin 26; BPDAI, the Bullous Pemphigoid Disease Area Index; NLR, neutrophil-to-lymphocyte ratio; MPO, myeloperoxidase; MBP, major basic protein; DES, dermal-epidermal separation; ELISA, enzyme-linked immunosorbent assay; DPP-4, dipeptidyl peptidase-4; PBMC, primary blood mononuclear cells; NHEK, normal human epidermal keratinocytes; NS, Not Significant.
