# Supplementary material for: Interleukin-26–DNA complexes promote inflammation and dermal-epidermal separation in a modified human cryosection model of bullous pemphigoid
Source: Front Immunol. 2022 Oct 10;13:1013382. doi: 10.3389/fimmu.2022.1013382 (PMC9599390; doi:10.3389/fimmu.2022.1013382)
Supplement: Supplementary file 1 [file Table_1.docx]

**Supplementary Table 1.** **Clinical characteristics of BP patients and healthy controls.**

|  | BP patients | Healthy controls |
| --- | --- | --- |
|  | n = 48 | n = 33 |
| Age, median (IQR) years | 67.5 (57.5- 74) | 66 (56- 72) |
| Sex, male/female | 22/26 | 14/19 |

BP; bullous pemphigoid, IQR; interquartile range
