# Supplementary material for: Interleukin-26–DNA complexes promote inflammation and dermal-epidermal separation in a modified human cryosection model of bullous pemphigoid
Source: Front Immunol. 2022 Oct 10;13:1013382. doi: 10.3389/fimmu.2022.1013382 (PMC9599390; doi:10.3389/fimmu.2022.1013382)
Supplement: Supplementary file 2 [file Table_2.docx]

**Supplementary Table 2**. **Sequences of the primers used for quantitative real-time PCR**

| Gene | Forward | Reverse |
| --- | --- | --- |
| MMP-9 | 5'-TGTACCGCTATGGTTACACTCG- 3′ | 5' -GGCAGGGACAGTTGCTTCT- 3′ |
| IL-1β | 5'-ATGATGGCTTATTACAGTGGCAA- 3′ | 5' -GTCGGAGATTCGTAGCTGGA- 3′ |
| IL-6 | 5' -ACTCACCTCTTCAGAACGAATTG- 3′ | 5' -CCATCTTTGGAAGGTTCAGGTTG- 3′ |
| GAPDH | 5' -GACAGTCAGCCGCATCTTCT- 3′ | 5' -GCGCCCAATACGACCAAATC- 3′ |
