# Supplementary figures and images for: Interleukin-26–DNA complexes promote inflammation and dermal-epidermal separation in a modified human cryosection model of bullous pemphigoid
Source: Front Immunol. 2022 Oct 10;13:1013382. doi: 10.3389/fimmu.2022.1013382 (PMC9599390; doi:10.3389/fimmu.2022.1013382)

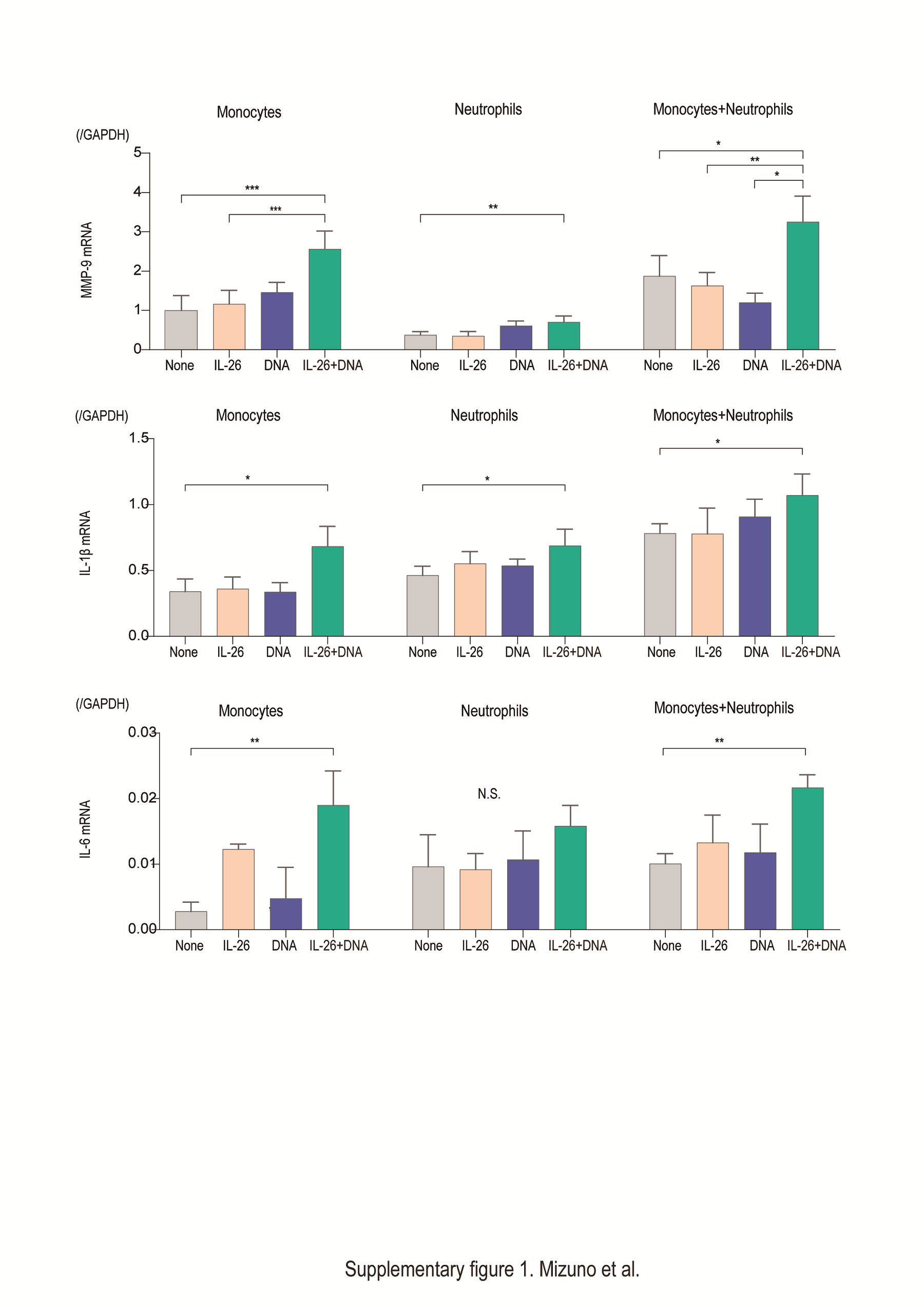

Supplement: Supplementary Figure 1 — Gene expression levels in monocytes alone, neutrophils alone, and co-cultured monocytes and neutrophils 24 hours after stimulation Cells were stimulated with/without 50 ng/mL of IL-26 and 10 µg/mL genomic DNA or with IL-26–DNA complexes for 24 hours. The relative mRNA expressions of MMP-9, IL-1β, and IL-6 were evaluated by quantitative real-time PCR. Data are presented as mean ± SD. *p < 0.05**p < 0.001***p < 0.0001. [file Image_1.tif]
